# Crystal structure of 2-hy­droxy-3-(prop-2-yn-1-yl)naphthalene-1,4-dione

**DOI:** 10.1107/S2056989018011647

**Published:** 2018-08-24

**Authors:** Isidório Raquel Geralda, Ottoni Flaviano Melo, Alves Ricardo José, Speziali Nivaldo Lúcio

**Affiliations:** a Departamento de Produtos Farmacêuticos, Faculdade de Farmácia, Universidade Federal de Minas Gerais, Avenida Antônio Carlos, 6627, Belo Horizonte Minas Gerais, CEP 31.270-901, Brazil; b Departamento de Física, Instituto de Ciências Exatas, Universidade Federal de Minas Gerais, Avenida Antônio Carlos, 6627, Belo Horizonte, Minas Gerais, CEP 31.270-901, Brazil

**Keywords:** crystal structure, naphtho­quinones, 2-hy­droxy-3-(prop-2-yn-1-yl)naphthalene-1,4-dione

## Abstract

The naphtho­quinone unit of the title compound, C_13_H_8_O_3_, is essentially planar, with an r.m.s. deviation of 0.013 Å for the non-H atoms. The essentially linear propargyl group is tilted by *ca* 113° relative to the naphtho­quinone plane. In the crystal, mol­ecules are linked *via* a pair of O—H⋯O hydrogen bonds, forming an inversion dimer. The dimers are further linked *via* pairs of C—H⋯O hydrogen bonds into a tape structure along [20]. No π–π stacking is observed in the present case as it could be expected for naphtho­quinone derivatives.

## Abstract 2

The naphtho­quinone unit in 2-hy­droxy-3-(prop-2-yn-1-yl)naphthalene-1,4-dione is essentially planar and the linear propargyl group is nearly perpendicular to the naphthalene ring system. In the crystal, O—H⋯O and C—H⋯O hydrogen bonds form an infinite tape structure.

## Chemical context   

Lawsone (2-hy­droxy­naphtalene-1,4-dione), **1**, shows prom­ising in the synthesis of analogues of atovaquone, **2**, an anti­malarial drug (Nixon *et al.*, 2013 ▸) also used in immunosuppressed patients affected by pneumonia caused by *Pneumocystis carinii* (Cirioni *et al.*, 1995 ▸; Comley *et al.*, 1995 ▸). Recent studies have shown that it can be also useful in the fight against cancer (Fiorillo *et al.*, 2016 ▸; Ashton *et al.*, 2016 ▸). Thus far unknown, 2-hy­droxy-3-(prop-2-yn-1-yl)naphthalene-1,4-dione (**3**) was obtained in a two steps one-pot procedure by reacting **1** with propargyl iodide, prepared *in situ* from propargyl bromide and potassium iodide. It opens the possibility for the synthesis of triazoles at the C3 position of **1** by [2 + 3] alkyne–azide 1,3-dipolar cyclo­addition enabling the preparation of 3-substituted lawsone derivatives with potential pharmacological activity, including atovaquone (**2**) analogues.
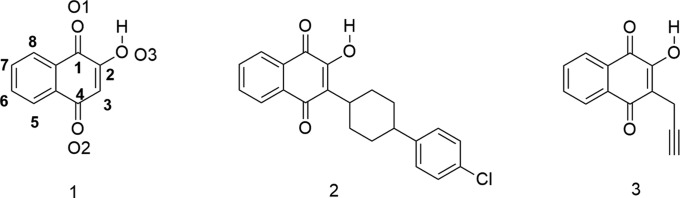


Treatment of **1** with a base leads to the formation of the corresponding enolate that can be *O*- or *C*-alkyl­ated depending on the nature of the counter-ion, reaction conditions and nature of the alkyl electrophile (Jordão *et al.*, 2015 ▸). When **1** was reacted with propargyl bromide and sodium carbonate in DMF the 2-*O*-propargyl derivative was obtained in 20% yield (Valença *et al.*, 2017 ▸). The 3-*C*-propargyl deriv­ative had not been described thus far. In view of the importance of acetyl­enic compounds for [2 + 3] alkyne–azide 1,3-dipolar cyclo­addition reactions, known as the *click* reaction, we decided to investigate the 2-*O*- *versus* 3-*C*-propargylation of **1**. The 3-*C*-propargyl derivative is considered to be an inter­esting inter­mediate for the synthesis of 3-triazolo analogues of atovaquone, **2**, and other bioactive 1,4-naphtho­quinones. After evaluating the influence of organic and inorganic bases, protic and aprotic solvents, alkyl­ating agents, temperature and reaction time, we obtained **3** in 28% yield. No product of *O*-alkyl­ation was observed in the reaction mixture.

## Structural commentary   

**Figure 1:**
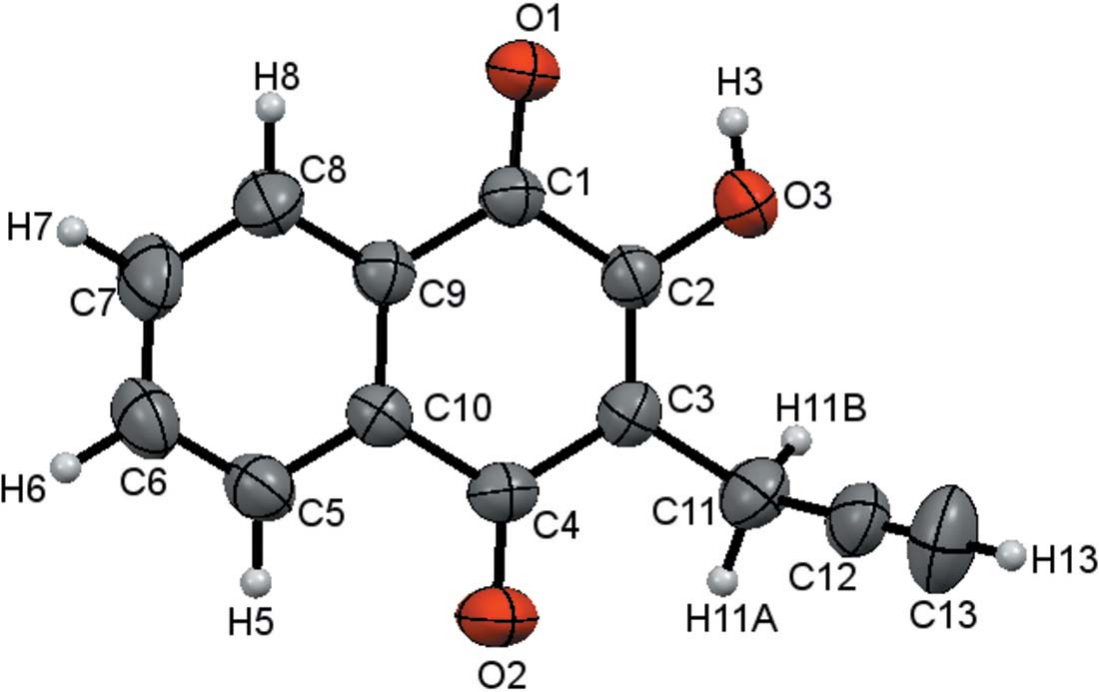
The mol­ecular structure of the title compound **3**. Displacement ellipsoids are drawn at the 50% probability level.

The molecular structure of the title compound, **3**, is shown in Fig. 1 ▸. The naphtho­quinone unit is essentially planar, with an r.m.s. deviation of 0.013 Å for the non-H atoms. The C—O bond lengths [C1—O1 = 1.2217 (18) Å, C2—O3 = 1.3412 (18) Å and C4—O2 = 1.2488 (19) Å] confirm the presence of 2-hy­droxy­naphthalene-1,4-dione in the crystalline state and are in agreement with the lengths found by Dekkers *et al.* (1996 ▸). The ^1^H and ^13^C NMR spectra and HMBC experiments confirm atoms C1 and C4 as carbonyls, as well as a hy­droxy group at C2. The propargyl group adopts a nearly perpendic­ular position [C3—C11—C12 = 112.70 (14)°] regarding the naphthalene ring system to avoid hindrance with the O2 and O3 atoms. The naphthoquinone ring system is characterized by the torsion angles C4—C3—C11—C12 = −100.96 (19)° and C2—C3—C11—C12 = 79.9 (2)°.

## Supra­molecular features   

**Figure 2:**
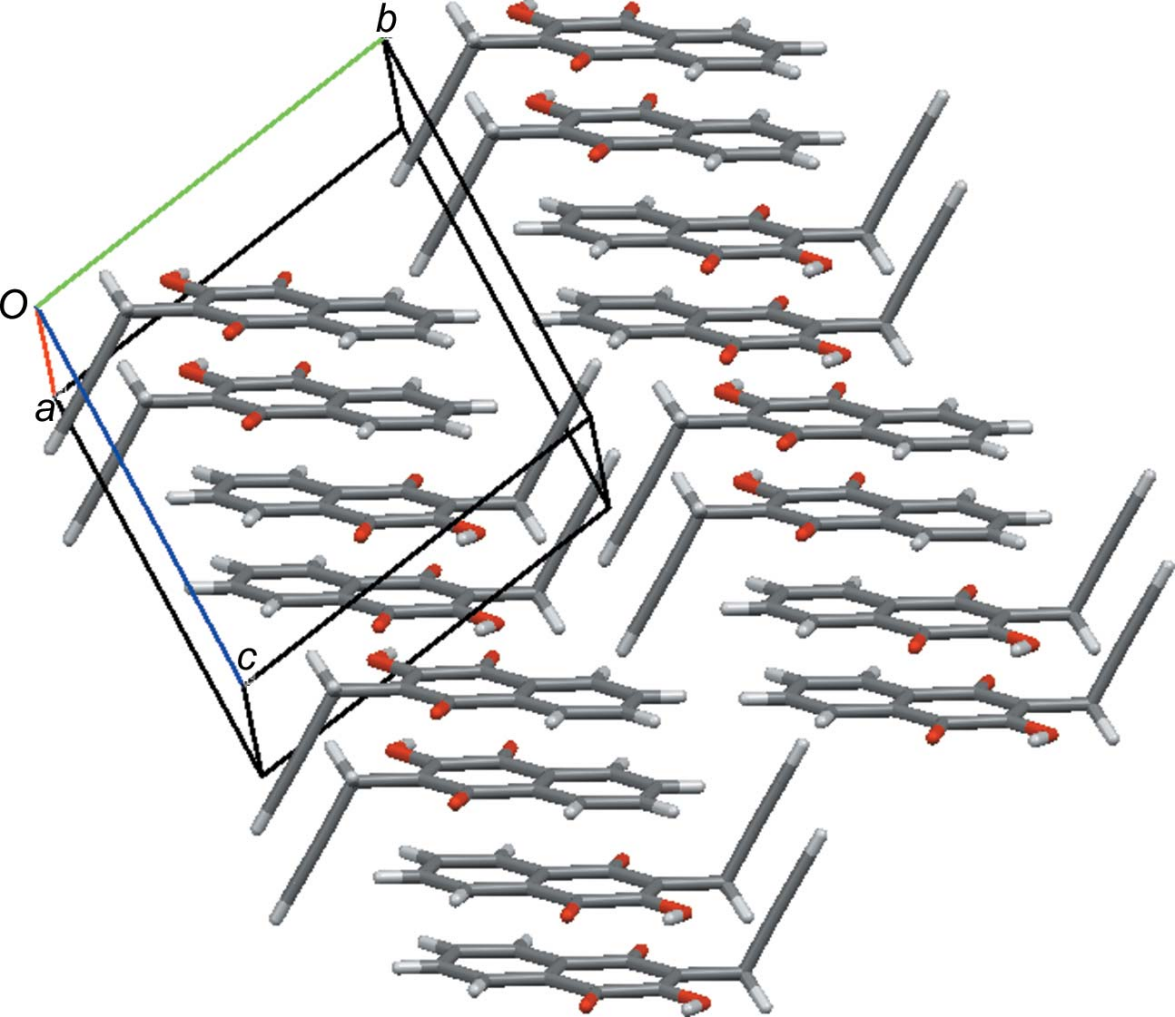
A packing diagram of the title compound.

In the crystal, O—H⋯O and C—H⋯O hydrogen bonds (O3—H3⋯O1^i^ and C5—H5⋯O2^ii^; symmetry codes as in Table 1 ▸) are responsible for an infinite tape structure running along [20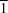]. All the naphtho­quinone units are arranged in a parallel manner with respect to each other, as shown in Fig. 2 ▸. π–π stacking inter­actions are expected for naphtho­quinone derivatives (Meyer *et al.*, 2003 ▸). However, this type of inter­action is not observed here, probably because of the C3 propargyl substituent.

## Database survey   

A search of the Cambridge Structural Database (CSD, Version 5.39, last update May 2018; Groom *et al.*, 2016 ▸) for 2-hy­droxy-naphthalene-1,4-dione revealed 40 structures and approximately 787 structures which possess the naphthalene-1,4-dione moiety. 2-Hy­droxy-3-(3-oxobut­yl)naphthalene-1,4-dione (Nasiri *et al.*, 2006 ▸) and 2-hy­droxy-3-(methyl-prop-1-en-1-yl)naphthalene-1,4-dione (Alcantara Emiliano *et al.*, 2016 ▸), compounds with structural similarity to the title compound, were also found. These compounds present a group linked to C3 with an angle nearly perpendicular to the naphtho­quinone ring.

## Synthesis and crystallization   

**Figure 3:**
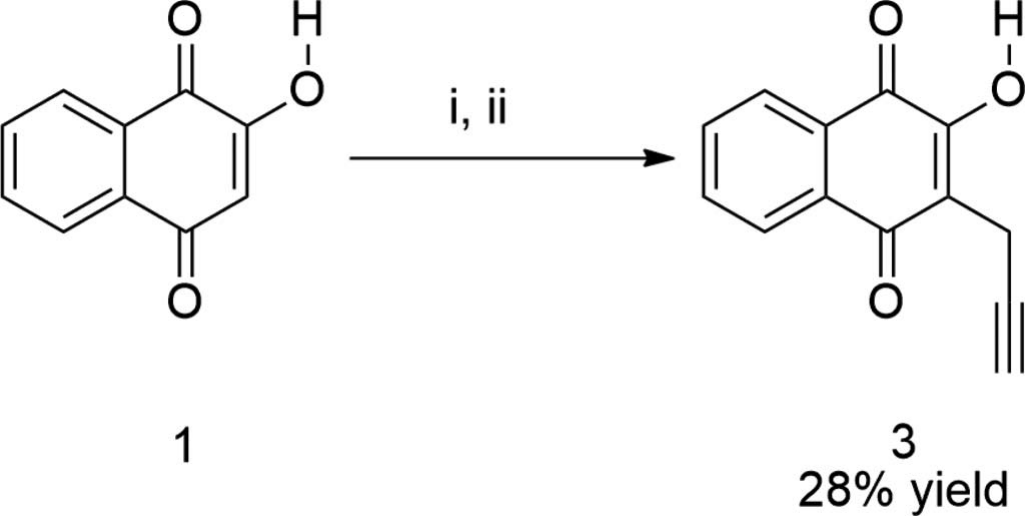
The synthetic scheme of the title compound, **3**; (i) propargyl bromide, sodium iodide and dry acetone, 0.5 h; (ii) diiso­propyl­ethyl­amine and *t*-BuOH/H_2_O, 353 K, 24 h.

The synthetic scheme is shown in Fig. 3 ▸. A mixture of propargyl bromide (0.75 ml, 4.47 mmol) and sodium iodide (1.30 g, 5.33 mmol) in dry acetone (3.5 ml) was stirred for 30 min at room temperature in a closed system. Then, a solution of lawsone (0.1 g, 2.4 mmol) and diiso­propyl­ethyl­amine (0.51 ml, 2.93 mmol) in a 2:1 (*v*/*v*) mixture of water/*tert*-butanol (24 ml) was added and the reaction mixture was stirred for a further 24 h at 353 K. The reaction was quenched with di­chloro­methane (*ca* 40 ml) and the heterogeneous mixture was transferred to a separatory funnel. The aqueous phase was separated and the organic layer was extracted with 1 mol l^−1^ hydro­chloric acid (3 × 40 ml) and water (3 × 40 ml). The organic layer was dried over anhydrous sodium sulfate and concentrated to dryness. The crude red solid product (0.45 g) was purified by column chromatography (silica) using a 99.5:0.5 (*v*/*v*) mixture of hexa­ne/*tert*-butanol containing 0.1% of acetic acid as eluent. Pure title compound was obtained in 28% yield (0.143 g, m.p. 396.7–397.2 K). Single crystals suitable for X-ray analysis were obtained by slow evaporation of a hexa­ne/*tert*-butanol solution (*ca* 0.5 mg ml^−1^) at room temperature. The infrared and NMR spectral data and corresponding spectra of **3** are available in the supporting information.

## Refinement   

Crystal data, data collection and structure refinement details are summarized in Table 2 ▸. C-bound H atoms were placed geometrically (C—H = 0.93–0.97 Å) and were refined as riding with *U*_iso_(H) = 1.2*U*_eq_(C). The O-bound H atom was located in a difference Fourier map and freely refined [O—H = 0.89 (3) Å].

## Supplementary Material

Crystal structure: contains datablock(s) I. DOI: 10.1107/S2056989018011647/is5498sup1.cif

Supporting information file. DOI: 10.1107/S2056989018011647/is5498Isup3.cml

The infrared and NMR spectroscopic data and corresponding spectra of <b>3</b>. DOI: 10.1107/S2056989018011647/is5498sup4.pdf

CCDC reference: 1862442

Additional supporting information:  crystallographic information; 3D view; checkCIF report

## Figures and Tables

**Table 1:** Hydrogen-bond geometry (Å, °)

*D*—H⋯*A*	*D*—H	H⋯*A*	*D*⋯*A*	*D*—H⋯*A*
O3—H3⋯O1^i^	0.89 (3)	2.06 (3)	2.8118 (19)	142 (3)
C5—H5⋯O2^ii^	0.93	2.49	3.231 (2)	137

Symmetry codes: (i) 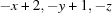; (ii) 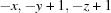.

**Table 2:** Experimental details

Crystal data	
Chemical formula	C_13_H_8_O_3_
*M*_r_	212.19
Crystal system, space group	Triclinic, *P* 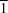
Temperature (K)	293
*a*, *b*, *c* (Å)	5.3695 (4), 9.5278 (8), 10.2972 (9)
α, β, γ (°)	96.814 (7), 93.432 (7), 102.977 (7)
*V* (Å^3^)	507.68 (8)
*Z*	2
Radiation type	Mo *K*α
μ (mm^−1^)	0.10
Crystal size (mm)	0.4 × 0.2 × 0.05

Data collection	
Diffractometer	Rigaku Xcalibur Atlas Gemini ultra
Absorption correction	Multi-scan (*CrysAlis PRO*; Rigaku OD, 2015 ▸)
*T*_min_, *T*_max_	0.720, 1.000
No. of measured, independent and observed [*I* > 2σ(*I*)] reflections	7946, 2508, 1563
*R*_int_	0.033
(sin θ/λ)_max_ (Å^−1^)	0.695

Refinement	
*R*[*F* ^2^ > 2σ(*F* ^2^)], *wR*(*F* ^2^), *S*	0.051, 0.147, 1.05
No. of reflections	2508
No. of parameters	149
H-atom treatment	H atoms treated by a mixture of independent and constrained refinement
Δρ_max_, Δρ_min_ (e Å^−3^)	0.21, −0.5

Computer programs: *CrysAlis PRO* (Rigaku OD, 2015 ▸), *SHELXT* (Sheldrick, 2015*a* ▸), *SHELXL2014* (Sheldrick, 2015*b*) and *OLEX2* (Dolomanov *et al.*, 2009 ▸ ▸).

## supplementary crystallographic information

### Crystal data

**Table:**

C_13_H_8_O_3_	*F*(000) = 220
*M**_r_* = 212.19	*D*_x_ = 1.388 Mg m^−^^3^
Triclinic, *P*1	Melting point = 396.8–397.5 K
*a* = 5.3695 (4) Å	Mo *K*α radiation, λ = 0.71073 Å
*b* = 9.5278 (8) Å	Cell parameters from 1721 reflections
*c* = 10.2972 (9) Å	θ = 3.2–28.7°
α = 96.814 (7)°	µ = 0.10 mm^−^^1^
β = 93.432 (7)°	*T* = 293 K
γ = 102.977 (7)°	Prism, colourless
*V* = 507.68 (8) Å^3^	0.4 × 0.2 × 0.05 mm
*Z* = 2	

### Data collection

**Table:**

Rigaku Xcalibur Atlas Gemini ultra diffractometer	2508 independent reflections
Radiation source: fine-focus sealed X-ray tube, Enhance (Mo) X-ray Source	1563 reflections with *I* > 2σ(*I*)
Graphite monochromator	*R*_int_ = 0.033
Detector resolution: 10.4186 pixels mm^-1^	θ_max_ = 29.6°, θ_min_ = 2.8°
ω scans	*h* = −7→7
Absorption correction: multi-scan (CrysAlis PRO; Rigaku OD, 2015)	*k* = −12→12
*T*_min_ = 0.720, *T*_max_ = 1.000	*l* = −13→14
7946 measured reflections	

### Refinement

**Table:**

Refinement on *F*^2^	Primary atom site location: dual
Least-squares matrix: full	Hydrogen site location: mixed
*R*[*F*^2^ > 2σ(*F*^2^)] = 0.051	H atoms treated by a mixture of independent and constrained refinement
*wR*(*F*^2^) = 0.147	*w* = 1/[σ^2^(*F*_o_^2^) + (0.0639*P*)^2^ + 0.058*P*] where *P* = (*F*_o_^2^ + 2*F*_c_^2^)/3
*S* = 1.05	(Δ/σ)_max_ < 0.001
2508 reflections	Δρ_max_ = 0.21 e Å^−^^3^
149 parameters	Δρ_min_ = −0.5 e Å^−^^3^
0 restraints	

### Special details

**Table:**

Geometry. All esds (except the esd in the dihedral angle between two l.s. planes) are estimated using the full covariance matrix. The cell esds are taken into account individually in the estimation of esds in distances, angles and torsion angles; correlations between esds in cell parameters are only used when they are defined by crystal symmetry. An approximate (isotropic) treatment of cell esds is used for estimating esds involving l.s. planes.

### Fractional atomic coordinates and isotropic or equivalent isotropic displacement parameters (Å^2^)

**Table:**

	*x*	*y*	*z*	*U*_iso_*/*U*_eq_	
O3	0.6461 (3)	0.33269 (14)	0.01419 (12)	0.0536 (4)	
H3	0.796 (5)	0.389 (3)	0.002 (3)	0.104 (10)*	
O1	0.9195 (2)	0.59305 (13)	0.12532 (12)	0.0518 (3)	
O2	0.0447 (3)	0.36386 (14)	0.31887 (13)	0.0647 (4)	
C1	0.7232 (3)	0.54469 (17)	0.17533 (15)	0.0388 (4)	
C2	0.5615 (3)	0.39989 (17)	0.11881 (15)	0.0403 (4)	
C3	0.3415 (3)	0.33844 (17)	0.16560 (15)	0.0419 (4)	
C4	0.2483 (3)	0.41556 (18)	0.27733 (16)	0.0431 (4)	
C5	0.3230 (4)	0.6358 (2)	0.44404 (17)	0.0511 (4)	
H5	0.1693	0.5951	0.4767	0.061*	
C6	0.4664 (4)	0.7695 (2)	0.50175 (19)	0.0586 (5)	
H6	0.4109	0.8182	0.5740	0.070*	
C7	0.6917 (4)	0.8311 (2)	0.4525 (2)	0.0642 (6)	
H7	0.7871	0.9218	0.4915	0.077*	
C8	0.7782 (3)	0.7597 (2)	0.34557 (18)	0.0530 (5)	
H8	0.9302	0.8021	0.3123	0.064*	
C9	0.6345 (3)	0.62361 (16)	0.28851 (15)	0.0389 (4)	
C10	0.4061 (3)	0.56178 (17)	0.33796 (15)	0.0397 (4)	
C11	0.1798 (4)	0.19019 (18)	0.10516 (18)	0.0536 (5)	
H11A	0.0054	0.1811	0.1290	0.064*	
H11B	0.1751	0.1833	0.0103	0.064*	
C12	0.2779 (4)	0.07067 (19)	0.14761 (17)	0.0552 (5)	
C13	0.3566 (5)	−0.0258 (2)	0.1781 (2)	0.0829 (7)	
H13	0.4196	−0.1030	0.2026	0.099*	

### Atomic displacement parameters (Å^2^)

**Table:**

	*U*^11^	*U*^22^	*U*^33^	*U*^12^	*U*^13^	*U*^23^
O3	0.0570 (8)	0.0492 (7)	0.0513 (7)	0.0084 (6)	0.0167 (6)	−0.0053 (5)
O1	0.0456 (7)	0.0531 (7)	0.0548 (7)	0.0048 (6)	0.0176 (6)	0.0060 (5)
O2	0.0540 (8)	0.0635 (9)	0.0699 (9)	−0.0031 (6)	0.0256 (7)	0.0043 (7)
C1	0.0372 (9)	0.0400 (8)	0.0405 (8)	0.0091 (7)	0.0057 (7)	0.0094 (6)
C2	0.0433 (9)	0.0397 (9)	0.0391 (9)	0.0119 (7)	0.0057 (7)	0.0047 (6)
C3	0.0437 (9)	0.0374 (8)	0.0430 (9)	0.0075 (7)	0.0018 (7)	0.0040 (7)
C4	0.0395 (9)	0.0443 (9)	0.0453 (9)	0.0061 (7)	0.0073 (7)	0.0110 (7)
C5	0.0499 (10)	0.0575 (11)	0.0468 (10)	0.0140 (8)	0.0121 (8)	0.0047 (8)
C6	0.0615 (12)	0.0620 (12)	0.0503 (10)	0.0185 (10)	0.0071 (9)	−0.0096 (8)
C7	0.0615 (13)	0.0533 (11)	0.0678 (13)	0.0076 (10)	0.0001 (10)	−0.0172 (9)
C8	0.0441 (10)	0.0488 (10)	0.0611 (11)	0.0041 (8)	0.0066 (8)	−0.0004 (8)
C9	0.0377 (9)	0.0390 (8)	0.0402 (8)	0.0101 (7)	0.0019 (7)	0.0046 (6)
C10	0.0389 (9)	0.0430 (9)	0.0380 (8)	0.0106 (7)	0.0049 (7)	0.0055 (7)
C11	0.0497 (10)	0.0458 (10)	0.0582 (11)	0.0004 (8)	0.0023 (9)	0.0006 (8)
C12	0.0671 (12)	0.0395 (10)	0.0515 (10)	0.0016 (9)	0.0070 (9)	−0.0039 (8)
C13	0.113 (2)	0.0483 (12)	0.0862 (16)	0.0257 (13)	−0.0048 (14)	0.0002 (11)

### Geometric parameters (Å, º)

**Table:**

O3—H3	0.88 (3)	C6—H6	0.9300
O3—C2	1.3426 (19)	C6—C7	1.375 (3)
O1—C1	1.2217 (18)	C7—H7	0.9300
O2—C4	1.2189 (19)	C7—C8	1.384 (3)
C1—C2	1.485 (2)	C8—H8	0.9300
C1—C9	1.473 (2)	C8—C9	1.392 (2)
C2—C3	1.340 (2)	C9—C10	1.390 (2)
C3—C4	1.465 (2)	C11—H11A	0.9700
C3—C11	1.521 (2)	C11—H11B	0.9700
C4—C10	1.499 (2)	C11—C12	1.458 (3)
C5—H5	0.9300	C12—C13	1.161 (3)
C5—C6	1.376 (3)	C13—H13	0.9300
C5—C10	1.381 (2)		
			
C2—O3—H3	106.5 (17)	C6—C7—C8	120.80 (18)
O1—C1—C2	118.90 (14)	C8—C7—H7	119.6
O1—C1—C9	123.40 (15)	C7—C8—H8	120.5
C9—C1—C2	117.69 (14)	C7—C8—C9	119.08 (17)
O3—C2—C1	115.90 (14)	C9—C8—H8	120.5
C3—C2—O3	120.71 (15)	C8—C9—C1	120.23 (15)
C3—C2—C1	123.38 (14)	C10—C9—C1	119.71 (14)
C2—C3—C4	119.88 (15)	C10—C9—C8	120.07 (15)
C2—C3—C11	122.10 (15)	C5—C10—C4	119.53 (15)
C4—C3—C11	118.01 (15)	C5—C10—C9	119.69 (15)
O2—C4—C3	121.03 (16)	C9—C10—C4	120.78 (14)
O2—C4—C10	120.40 (15)	C3—C11—H11A	109.1
C3—C4—C10	118.56 (14)	C3—C11—H11B	109.1
C6—C5—H5	119.8	H11A—C11—H11B	107.8
C6—C5—C10	120.37 (17)	C12—C11—C3	112.70 (14)
C10—C5—H5	119.8	C12—C11—H11A	109.1
C5—C6—H6	120.0	C12—C11—H11B	109.1
C7—C6—C5	119.98 (17)	C13—C12—C11	178.3 (2)
C7—C6—H6	120.0	C12—C13—H13	180.0
C6—C7—H7	119.6		
			
O1—C1—C2—O3	−0.4 (2)	C4—C3—C11—C12	−100.96 (19)
O1—C1—C2—C3	−179.15 (16)	O2—C4—C10—C5	2.0 (3)
C9—C1—C2—O3	178.72 (14)	O2—C4—C10—C9	−177.93 (16)
C9—C1—C2—C3	−0.1 (2)	C3—C4—C10—C5	−179.45 (16)
O1—C1—C9—C8	−1.0 (2)	C3—C4—C10—C9	0.7 (2)
O1—C1—C9—C10	178.81 (15)	C10—C5—C6—C7	1.0 (3)
C2—C1—C9—C8	179.98 (14)	C6—C5—C10—C4	179.40 (17)
C2—C1—C9—C10	−0.2 (2)	C6—C5—C10—C9	−0.7 (3)
O3—C2—C3—C4	−178.09 (15)	C5—C6—C7—C8	−0.5 (3)
O3—C2—C3—C11	1.0 (2)	C6—C7—C8—C9	−0.4 (3)
C1—C2—C3—C4	0.7 (2)	C7—C8—C9—C1	−179.48 (16)
C1—C2—C3—C11	179.77 (15)	C7—C8—C9—C10	0.7 (3)
C2—C3—C4—O2	177.64 (16)	C1—C9—C10—C4	−0.1 (2)
C2—C3—C4—C10	−0.9 (2)	C1—C9—C10—C5	−180.00 (16)
C11—C3—C4—O2	−1.5 (2)	C8—C9—C10—C4	179.73 (15)
C11—C3—C4—C10	179.92 (15)	C8—C9—C10—C5	−0.2 (2)
C2—C3—C11—C12	79.9 (2)		

### Hydrogen-bond geometry (Å, º)

**Table:**

*D*—H···*A*	*D*—H	H···*A*	*D*···*A*	*D*—H···*A*
O3—H3···O1^i^	0.89 (3)	2.06 (3)	2.8118 (19)	142 (3)
C5—H5···O2^ii^	0.93	2.49	3.231 (2)	137

Symmetry codes: (i) −*x*+2, −*y*+1, −*z*; (ii) −*x*, −*y*+1, −*z*+1.
