# Supplementary material for: Crystal structure of 2-hy­droxy-3-(prop-2-yn-1-yl)naphthalene-1,4-dione
Source: Acta Crystallogr E Crystallogr Commun. 2018 Aug 24;74(Pt 9):1319–21. doi: 10.1107/S2056989018011647 (PMC6127683; doi:10.1107/S2056989018011647)
Supplement: Supplementary file 3 [file e-74-01319-sup4.pdf]

SUPPORTING INFORMATION

1. Spectral data for compound 3

Fourier transform IR (FT—IR) spectrum was measured in the range 4000 - 650 cm^{-1} on a Perkin-Elmer FT—IR spectrophotometer Spectrum One. The spectrum shows the characteristic absorption bands of the main functional groups for title compound at IR (ν_{max}/cm^{-1}): 3340 (O—H), 3298, 3264 (C—H alkyne), 2955, 2922 (C—H sp^3), 2120 (C≡C, terminal alkyne), 1645 (C═O quinone), 1270, 1225, 1045 (C—O).

The NMR spectra were obtained in a Bruker Advance DRX 400 NMR spectrometer

^{1}H NMR(*δ*/ppm, *J*/Hz, 400 MHz, $CDCl_3$) 8.14 (dd, 1H $J_{5,6}$ 7.6 Hz, $J_{5,7}$ 1.2 Hz, H-5), 8.13 (dd, 1H, $J_{8,7}$ 7.6 Hz, $J_{8,6}$ 1.2 Hz, H-8), 7.76 (td, 1H, $J_{6,5}$ 7.6 Hz, $J_{6,7}$ 7.6 Hz, $J_{6,8}$ 1.2 Hz, H-6), 7.68 (td, 1H, $J_{7,6}$ 7.6 Hz, $J_{7,8}$ 7.6 Hz, $J_{7,5}$ 1.2 Hz, H-7), 7.58 (s, 1H, **H**O), 3.51 (d, 2H, $J_{11,13}$ 2.8 Hz, H-11), 1.97 (t, 1H, $J_{13,11}$ 2.8 Hz, H-13).

^{13}C NMR(*δ*/ppm, 100 MHz, $CDCl_3$): 183.4 (C-4), 181.5 (C-1), 153.5 (C-3),135.4(C-6), 133.4 (C-7), 132.9 (C-10), 129.5 (C-9), 127.2 (C-5), 126.6 (C-8),118.5 (C-2), 80.2 (C-12), 68.5 (C-13), 13.0 (C-11)

2. Spectra of compound 3

Infrared spectrum (FT-IR) of 3 in the range 4000-650 cm^{-1}

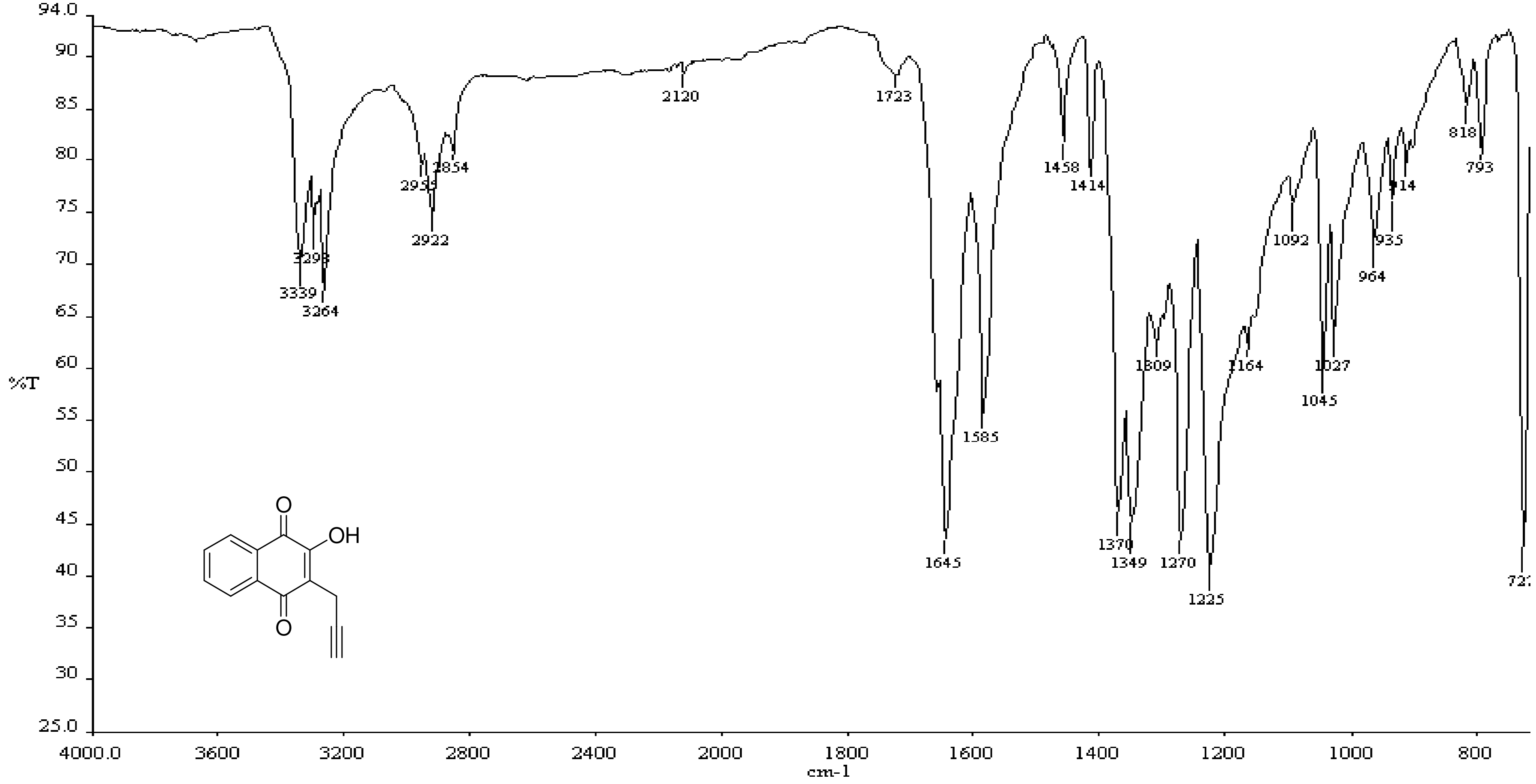

^{1}H NMR spectrum ($CDCl_3$, 400 MHz) of 3

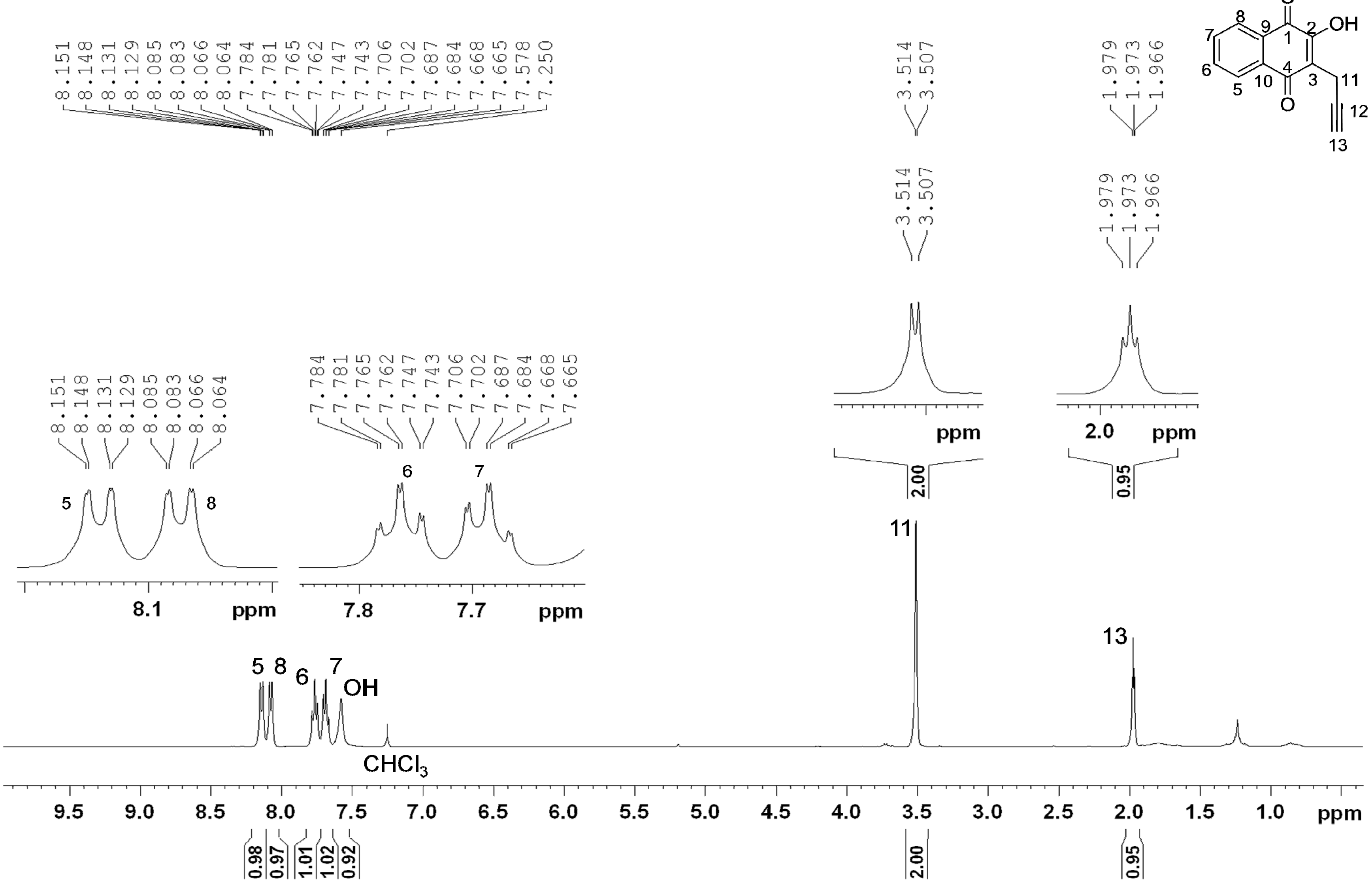

^{13}C NMR spectrum ($CDCl_3$, 100 MHz) of 3

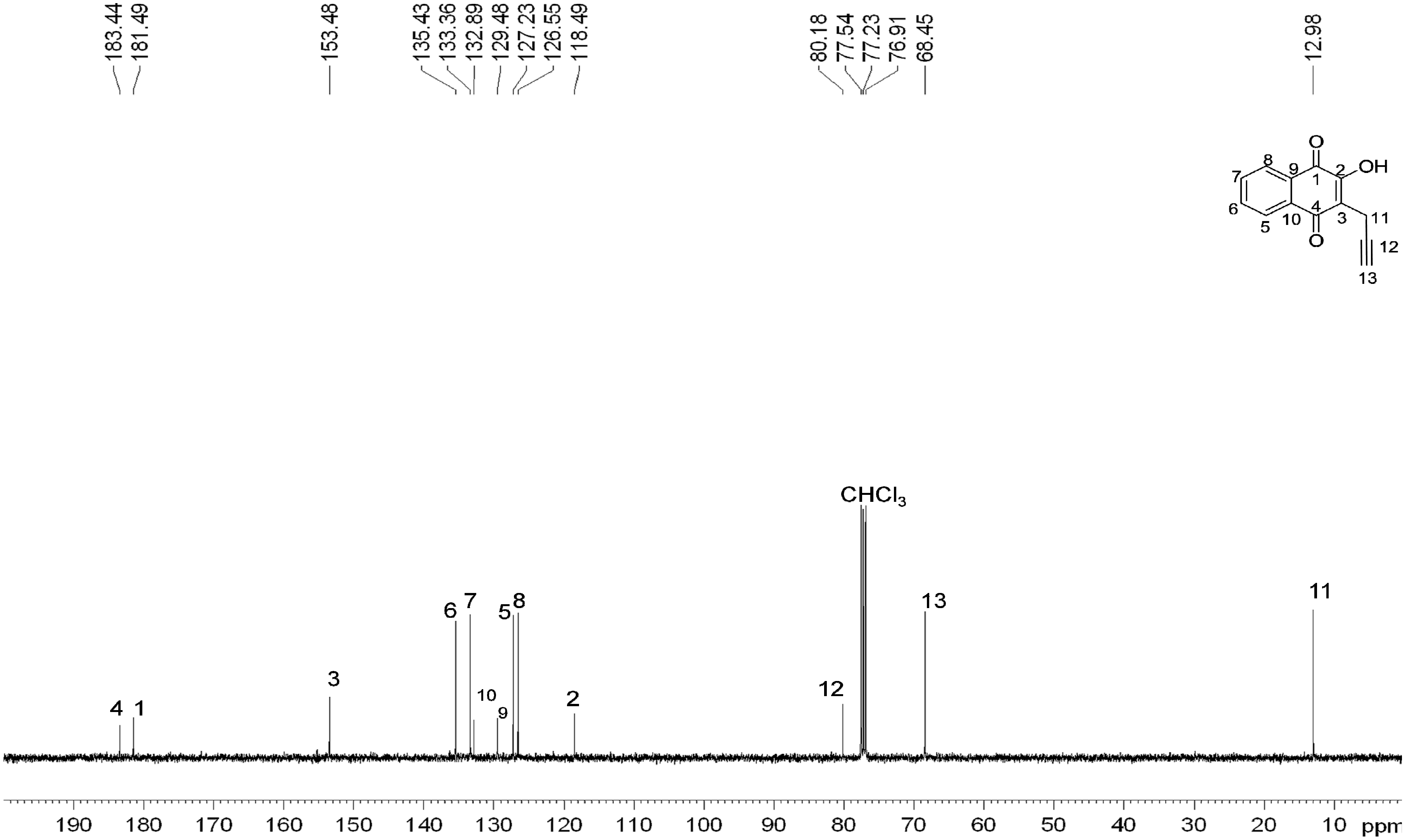

DEPT sub-spectrum ($CDCl_3$, 100 MHz) of 3

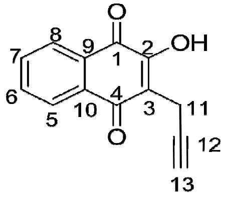

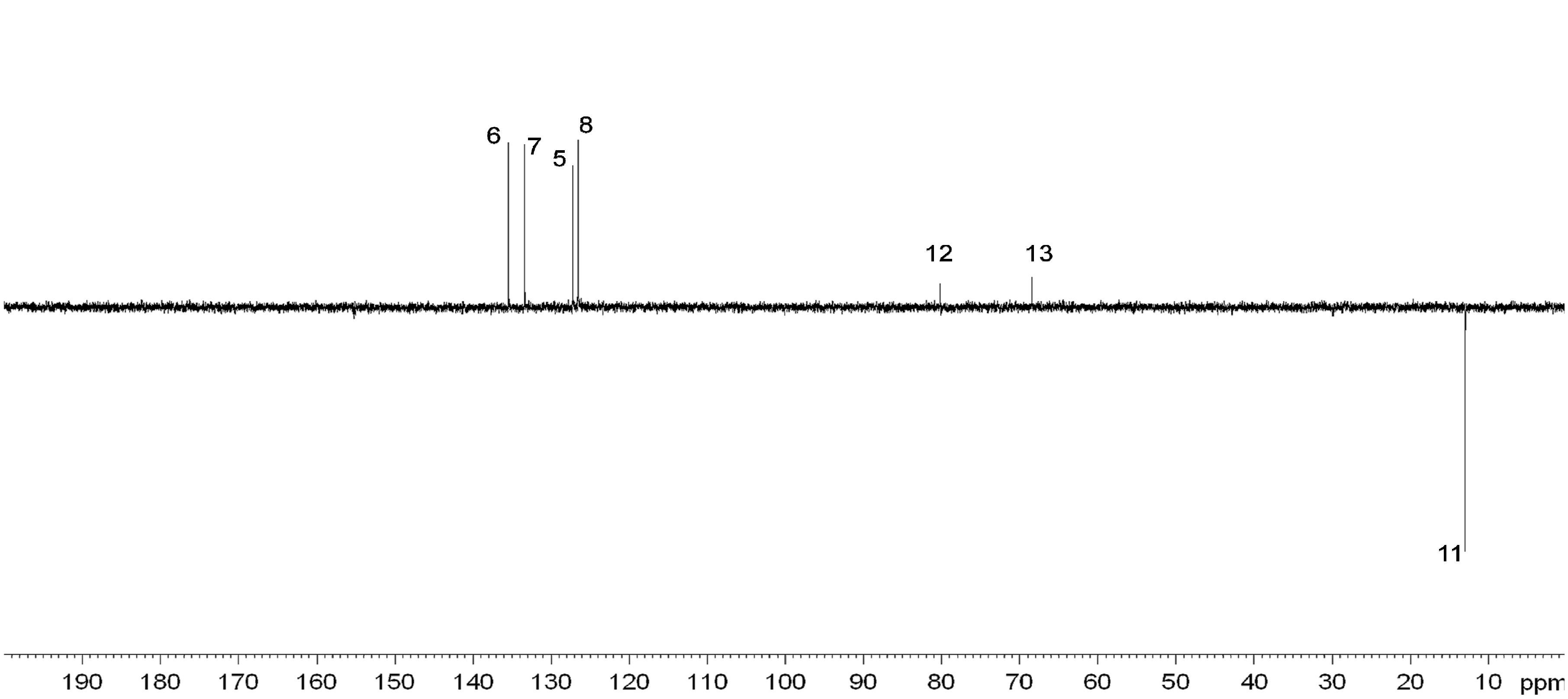

Cosy spectrum ($CDCl_3$, 400 MHz) of 3

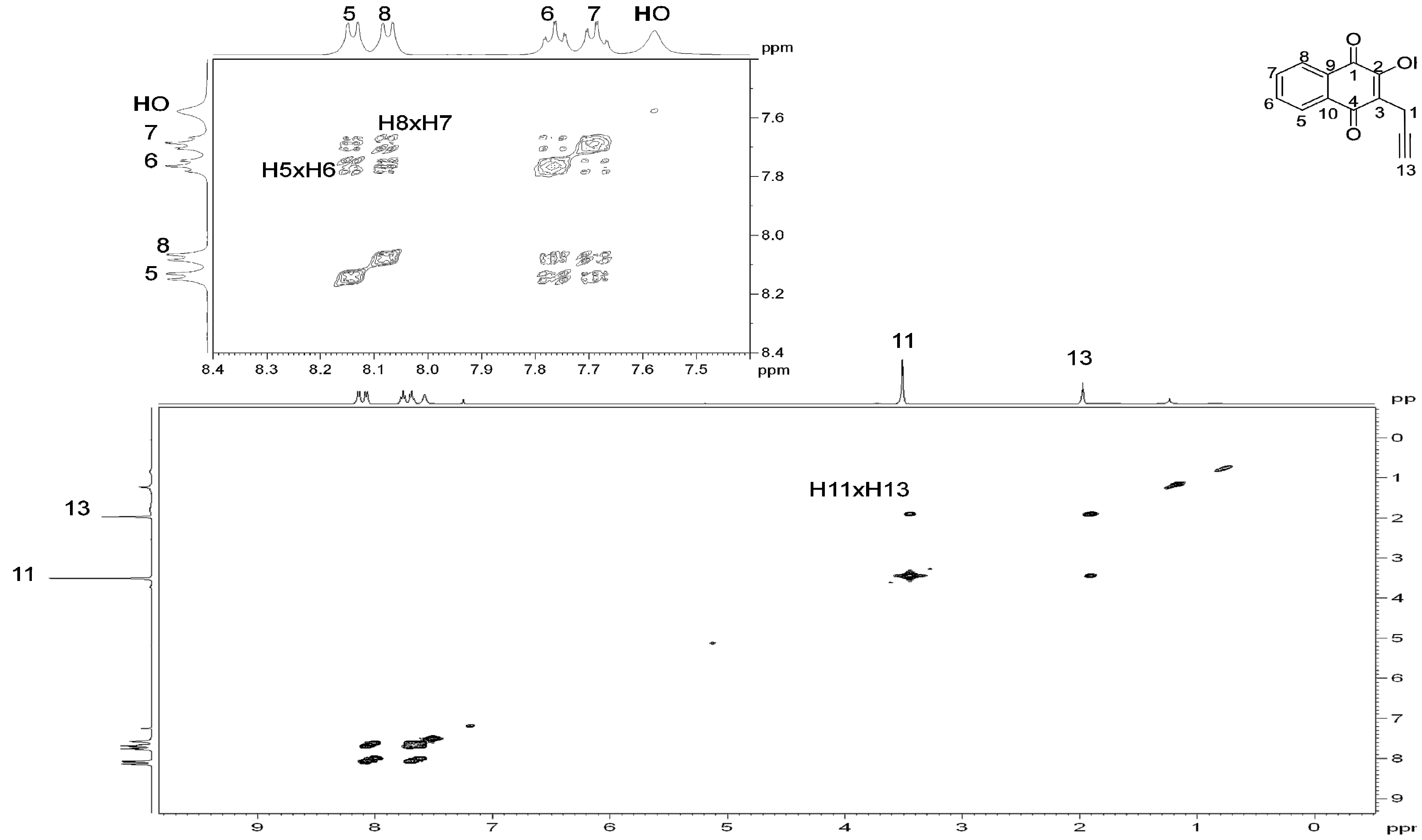

HSQC spectrum ($CDCl_3$) of 3

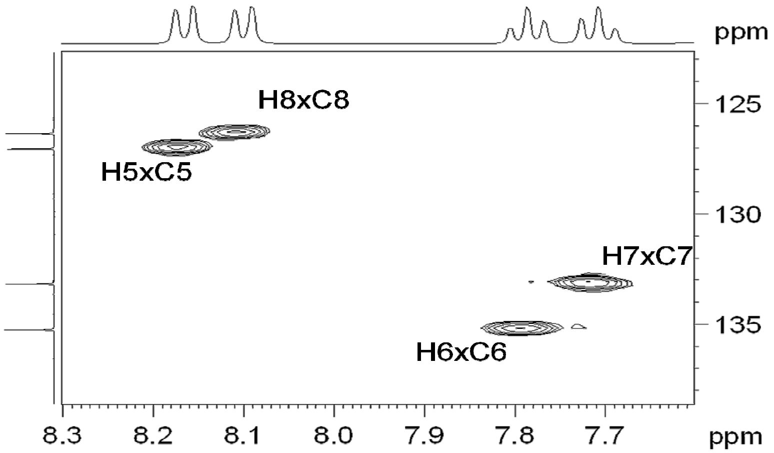

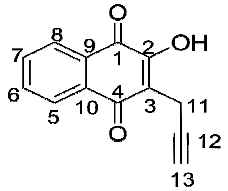

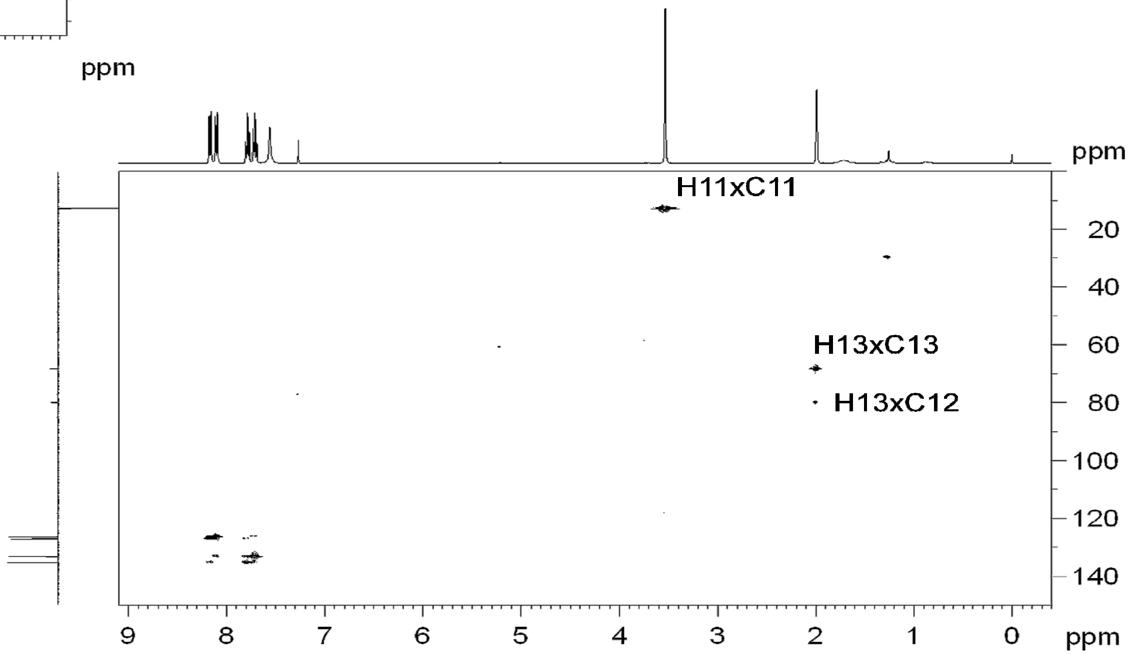

HMBC of 3

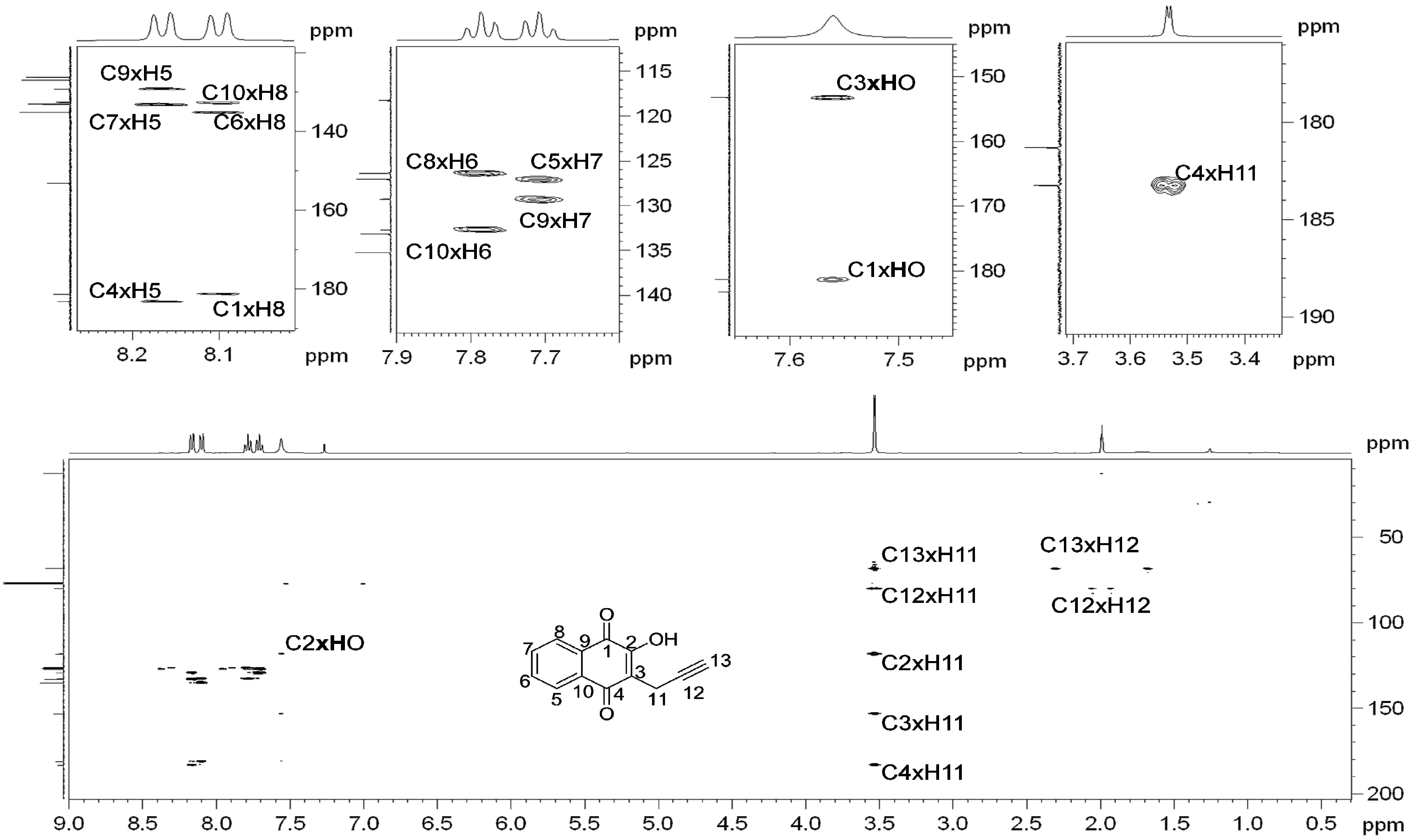

C9xH5
C10xH8
C7xH5
C6xH8
C4xH5
C1xH8
C8xH6
C5xH7
C9xH7
C10xH6
C3xHO
C1xHO
C4xH11
C2xHO
C13xH11
C12xH11
C2xH11
C3xH11
C4xH11
C13xH12
C12xH12
OH
ppm
